# Differential Stress Transcriptome Landscape of Historic and Recently Emerged Hypervirulent Strains of *Clostridium difficile* Strains Determined Using RNA-seq

**DOI:** 10.1371/journal.pone.0078489

**Published:** 2013-11-07

**Authors:** Joy Scaria, Chunhong Mao, Jenn-Wei Chen, Sean P. McDonough, Bruno Sobral, Yung-Fu Chang

**Affiliations:** 1 Department of Population Medicine and Diagnostic Sciences, Cornell University, Ithaca, New York, United States of America; 2 Virginia Bioinformatics Institute, Virginia Tech, Blacksburg, Virginia, United States of America; 3 Department of Biomedical Sciences, Cornell University, Ithaca, New York, United States of America;; Institute Pasteur, France

## Abstract

*C. difficile* is the most common cause of nosocomial diarrhea in North America and Europe. Genomes of individual strains of *C. difficile* are highly divergent. To determine how divergent strains respond to environmental changes, the transcriptomes of two historic and two recently isolated hypervirulent strains were analyzed following nutrient shift and osmotic shock. Illumina based RNA-seq was used to sequence these transcriptomes. Our results reveal that although *C. difficile* strains contain a large number of shared and strain specific genes, the majority of the differentially expressed genes were core genes. We also detected a number of transcriptionally active regions that were not part of the primary genome annotation. Some of these are likely to be small regulatory RNAs.

## Introduction

*Clostridium difficile* is a toxin producing anaerobic bacillus and is the leading cause of hospital associated diarrhea in North America and Europe[1,2]. Clinical presentation of *C. difficile* infection (CDI) ranges from asymptomatic colonization, mild diarrhea, severe pseudomembranous colitis, paralytic ileus, to sepsis and death[3]. Advanced age, prolonged hospitalization, antibiotic use and acid suppression therapy are some of the risk factors for CDI[3,4].The mortality rate is more than 80% in fulminant cases requiring colectomy[5]. *C. difficile* produces two toxins, toxin A and toxin B, which are responsible for most of the damage caused to the host[6,7]. 

During the last decade, there has been a significant increase in the rate of CDI across the United States, Canada, and Europe[8-10].Emergence of more virulent strains and changes in antibiotic treatment regimens are some of the established causes of the increase in CDI [11,12]. Some of these strains, which have the capacity to produce more severe colitis and mortality, have been termed as hypervirulent [13]. These strains belong to PCR ribotype 027[13]. Ribotype 027 strains are also characterized as toxinotype III, North American pulsed field gel electrophoresis type1 (NAP1) and restriction endonuclease analysis group BI and in contrast to historical control strains, are fluoroquinolone resistant[10]. In the recent years, *C. difficile* infection in the community setting has increased [14]. It is also causing a significant number of infections in food animals[15]. Recent studies have shown the possibility of foodborne transmission of *C. difficile*, which might explain the spread of *C. difficile* infections in the general community [16]. 

Comparative genomic studies have revealed that the genome conservation in *C. difficile* is very low and this is a major contributing factor in the outcome of infection by a given strain [17-19]. The number of core genes in *C. difficile* is estimated to be less than 20% of the pangenome[20]. This variation in the genome content could enable *C. difficile* strains to respond differently to environmental changes. A number of functional genomics studies have been conducted to indentify the differential gene network that controls *C. difficile* response to environmental changes[21-24]. However, all these studies are based *C. difficile* 630, a strain isolated in 1985 from Zurich, Switzerland. Genome of *C. difficile* 630 is the most commonly used reference for functional genomics studies as it was the first strain to be sequenced [17]. Another reason for using *C. difficile* 630 as reference is that microarray based studies require a high quality complete genome for probe design. 

Recent advances in new generation sequencing platforms have revolutionized microbial genomics. Genome sequencing using new generation sequencing platforms has resulted in sequencing of several *C. difficile* strains from different continents that are old as well as newer hypervirulent isolates[20,25-27]. These comparative genomics studies have revealed that when compared to historic strains (isolated in the 1980s), new hypervirulent strains (isolated after 2000) have undergone several genome changes that result in the hypervirulent phenotype[12,25]. The development of transcriptional profiling using RNA sequencing (RNA-seq) also offers vast improvements over microarray based transcriptional profiling. In order to determine how historic and recently emerged *C. difficile* strains respond to environmental changes we used RNA-seq to compare the transcriptome of four *C. difficile* strains after subjecting them to physiologically relevant *in vitro* stress conditions. In the first condition, cells were shifted from a rich medium [Brain heart infusion broth (BHI)] to a poor medium [Basal defined medium (BDM)] [28] with supplementation of 0.5% sucrose. It has been reported that the presence of glucose and other easily metabolizable carbon sources in the growth medium suppress production of toxin A and toxin B[29,30]. The shift from BHI to BDM was designed to induce multiple nutritional changes and to analyze the impact of those changes in *C. difficile* pathways. Osmotic shock (shift from BHI to BHI supplemented with 1.5% NaCl) was chosen as the second test condition because *C. difficile* has been shown to have enhanced host cell adherence following osmotic shock[31]. Since adhesion and colonization of animal tissue by bacteria is important in establishing infection, it is probable that without attachment, *C. difficile* cannot colonize and will be quickly removed by non-specific host defense mechanisms which include intestinal peristalsis, mucosal cell exfoliation and intestinal mucins[32,33].

Two strains in this comparison were isolated in the 1980s (CD630 and CD196), and two were hypervirulent strains isolated after 2000 (R20291 and QCD_32g58). Although s *C. difficile* 630 is not a ribotype 027 strain, we included this strain in the comparisons as it is the only strain that was sequenced using Sanger method and to enable cross comparison with the previously reported functional genomics studies. Consistent with the large genome diversity in *C. difficile*, our transcriptome sequencing results show that differentially expressed core genes in various strains are not identical. We also report expression of several novel transcripts that are not part of the primary genome annotation.

## Materials and Methods

### *C. difficile* strains and culturing

We selected four *C. difficile* strains for transcriptomic comparisons. The characteristics of these strains are given in Table 1. Bacterial culturing was performed inside a Bactron IV anaerobic chamber (Shel Lab, Cornelius, OR). The chamber was filled and purged with an anaerobic gas mixture (10% CO2, 85% N2, 5% H2). A palladium catalyst was used in the chamber to remove any trace of oxygen. All materials used in the anaerobic chamber were pre-reduced. Spores of *C. difficile* strains CD630, CD196, QCD_32g58 and R20291 were streaked on brain-heart infusion (BHI) agar plates containing 0.1% L-cysteine and taurocholate. The plates were incubated overnight at 37°C. Single colonies from these plates were then used inoculate pre-reduced BHI broth and were incubated at 37°C overnight. Fresh BHI broth was then inoculated by transferring 1% overnight culture. Cultures were incubated at 37°C until the OD600 reached between 0.4 - 0.5. Bacteria were then collected by centrifugation at 2,000 x g for 5 minutes. These cells were then shifted to two physiologically relevant *in vitro* conditions. In the first condition, cells were subjected to nutrient change by shifting to an equal volume of Basal defined medium (BDM)[28] with supplementation of 0.5% sucrose. This shift was designed to induce multiple nutritional changes and to analyze the impact of those changes in *C. difficile* pathways. In the second condition, cells were subjected to osmotic shock by shifting to an equal volume of BHI supplemented with 1.5% NaCl. The same number of cells was transferred to fresh BHI as the control group. After incubating for 1 hour at 37°C, twice the volume of RNAprotect bacteria reagent (Qiagen, Valencia, CA) was added to the cultures to halt transcription and RNA degradation, and cells were collected by centrifugation at 2,000 x g for 10 minutes.All experiments were performed as two biological replicates.

**Table 1:** Characteristics of *C. difficile* strains used in this study.

**Strain name**	**Ribotype**	**Isolation year**	**Isolation country**	**Hypervirulence**
CD630	13	1982	Switzerland	no
CD196	27	1985	France	no
QCD-32g58	27	2004	Canada	yes
R20291	27	2006	UK	yes

### Isolation of total RNA

Total RNA extraction was performed with TRIzol/RNeasy hybrid RNA extraction protocol. Briefly, the bacterial pellets were re-suspended with 1 ml of TRIzol reagent and were transferred to 2 ml sterile screw-cap microcentrifuge tube. Then 0.5ml of sterile RNase-free 0.1 mm zirconia beads was added to each tube. Cells were homogenized and lysed by bead beating four times in a Mini Bead-Beater (BioSpecProducts, Inc., Bartlesville, OK) for 30 seconds with a gap of 30 seconds. After the chloroform extraction, the aqueous phase was transferred to a 1.5 ml sterile RNase-free micro-centrifuge tube and mixed with an equal volume of 100% ethanol (Sigma). This mix was loaded into an RNeasy column (Qiagen kit) and centrifuged for 30 seconds at 8,000 x g. Washing of the column, DNA digestion and elution steps were performed following the standard Qiagen protocol. Integrity of isolated RNA was estimated using Agilent Bioanalyzer 2100. Only those samples with an RNA Integrity Number (RIN) >9 were used for RNA sequencing.

### cDNA library synthesis and sequencing

Ribo-Zero™ rRNA Removal Kit ( Epicentre® Biotechnologies, Madison, WI, USA) was used to purify mRNA from 10µg total RNA. First strand cDNA was synthesized using SuperScript® III (Invitrogen). The second-strand cDNA was synthesized using RNase H (Invitrogen) and DNA polymerase I (New England BioLabs). Then the cDNA libraries were prepared using the Illumina Paired-end Genomic DNA Sample Prep kit (Illumina) following the manufacturer's protocol. For each sample, two libraries were prepared from biological replicates. Each library was then loaded onto flow cell channels of the Illumina High-seq 2000 platform for paired-end 90 bp × 2 sequencing. The average insert size for the paired-end libraries was 200 bp (from 180 to 220 bp). For each strain, six paired-end cDNA libraries were constructed. Therefore twenty four libraries were sequenced across all strains. 

### RNA-Seq data analysis

For data processing, we used a customized RNA-Seq data analysis pipeline developed at Virginia Bioinformatics Institute (VBI) by combining open source programs. Briefly, the quality of the raw sequence reads was checked using the FastQC program (http://www.bioinformatics.bbsrc.ac.uk/projects/fastqc). The processed reads were then aligned using Bowtie version 0.12.7 [34] to the corresponding *C. difficile* reference genome. Read alignments with mapping quality score (MAPQ) < 10 were removed. Cufflinks software package version 1.3.1 [35] was used to assemble transcripts and estimate the relative abundances of the transcripts. Transcript expression levels are estimated as Fragments Per Kilobase per Million mapped reads (FPKM). Cuffdiff [36], a component of Cufflinks was used to calculate transcript expression levels. When compared to control condition, genes with log_2_ ratio ≥ 1.5 and FDR- adjusted *p* value less than or equal to 0.05 were considered as differentially expressed. The processed data and raw files from this experiment have been submitted to NCBI Gene expression Omnibus (GEO) under the accession # GSE50497 and NCBI short read archive (SRA) under the accession # SRP029366.

### Novel gene discovery

Cufflinks program provides reference annotation based assembly and seeks to build upon available information about the transcriptome of an organism to find novel genes and isoforms [35]. Cufflinks output includes all annotated reference transcripts and any novel genes and isoforms that are assembled. The novel gene transcripts identified by the pipeline can be novel small RNA genes or unannotated CDS. We performed the following steps to categorize these novel transcripts. First, these transcripts were used to search the Rfam database for sRNA matches. Rfam is a comprehensive database containing families of structural RNAs, including non-coding RNA genes as well as cis-regulatory RNA elements[37]. It incorporates literature curated known sRNAs and uses them as seeds to predict sRNAs for sequenced genomes using *covariance* model[37]. In the second step, the new transcripts were searched against the non-redundant (nr) database using BLASTX to check for any protein coding gene hits. Finally, we used ORF Finder program to verify whether any transcripts are potentially protein coding genes. Assembled transcripts with no BLASTX hits and no ORF assignment were considered as sRNAs. 

### Functional analysis of differentially expressed genes

Since it is well established that *C. difficile* strains are known to be highly divergent [18-20], we classified genes in each strain into core, shared and unique categories using OrthoMCL program [38]. We then combined this gene classification with gene expression level data to obtain a comparison of these genes across all strains. Pathologic program in Pathwaytools v 16.0[39] was used to reconstruct the metabolic pathways of strains QCD_32g58, CD196 and R20291. A previously curated high quality pathway annotation for strain CD630 by our group [23] was used to remove false positive pathway predictions in these strains. Omics viewer [40] was used to map the differentially expressed genes onto cellular pathways and to compare differentially expressed pathways across strains. For identifying how gene interaction networks adjust to the stress conditions tested, the differentially expressed genes were mapped to the systems level gene interaction network of *C. difficile*. The base interaction data for this analysis was obtained from STRING database[41]. Complete interaction data from STRING v 9.0 was downloaded and *C. difficile* specific interaction data was then extracted using custom Linux shell scripts. The interaction data in STRING database includes both experimental as well as predicted interactions and each interaction is assigned a confidence score. We then selected interactions with confidence score of 400 or above. These would represent medium and high confidence interactions in *C. difficile*. This interaction network was then imported into Cytoscape [42] for visualization and overlaying of transcriptomic expression data. 

### qRT-PCR

We used qRT-PCR to verify the expression levels of selected genes. Following the manufacturer’s instructions, 3.0 µg of total RNA isolated from each stress condition was converted to cDNA by using SuperScript III reverse transcriptase (Invitrogen) with random hexamers. The real-time reaction mixture included 12.5 ng cDNA, 200 nM of each of both forward and reverse primers, and 1X SYBR GreenER qPCR SuperMix (Invitrogen). Primers used in this study are listed in the File S1. qPCR was performed in 96-well optical plates using the AB 7500 Real-Time PCR System instrument and software (Invitrogen) and was analyzed by the method previously reported by our group[43]. 

## Results

### Genome coverage

*C. difficile* is an unusual species because the number of conserved genes in a given strain is very low and many of the strains contain a very high number of genes that are unique to that strain[19,20]. It has also undergone very rapid evolution in the last two decades by acquiring several new genes [25]. To understand how historic and recently emerged *C. difficile* respond to physiological stress, two historical (CD630 and CD196) and two recently emerged (QCD-32g58 and R20291) strains were subjected to nutrient shift and osmotic shock. Gene expression under these conditions was determined using RNA-seq and these results were compared to gene expression levels during growth in BHI (control condition). A total of 24 samples from these conditions were used for paired-end bi-directional Illumina sequencing. Illumina sequence files were converted to Sanger fastq format and rRNAs were filtered. The quality of the sequence data was checked using the FastQC program. The sequence reads were 90 nucleotides in length and the total number of reads per sample was ~ 26.6 million on average. The filtered RNA-Seq sequence reads from the 24 samples were aligned to their corresponding *C. difficile* reference genome using Bowtie. For all samples analyzed, 88-95% of reads were mapped with MAPQ greater than or equal to 10. The transcript expression levels were estimated as FPKM using Cufflinks[44]. The number of genes expressed (FPKM>0) was calculated for each sample. There was very high correlation between samples when the number of expressed genes was compared between biological replicates (Table 2). We used PATRIC [45] annotations of the *C. difficile* genomes as references. In this analysis, expression of more than 90% CDS was detected under the three conditions combined (Table 3). 

**Table 2:** Spearman correlation between biological replicates of RNA-seq datasets.

**Strain**	**Control A vs B**	**BDM A vs B**	**salt A vs B**
CD630	0.968	0.960	0.976
CD196	0.970	0.963	0.977
QCD-32g58	0.956	0.953	0.959
R20291	0.961	0.947	0.959

**Table 3:** Genes expressed in each strain and condition.

**Strain Name**	**Total number of genes**	**Number of genes not expressed**			**% of expressed genes**
		**Control**	**Nutrient shift**	**Osmotic shock**	
CD630	3858	274	229	255	94.06
CD196	3669	164	173	158	95.69
QCD-32g58	4224	444	456	421	90.03
R20291	3754	209	224	191	94.91

During nutrient shift, *C. difficile* strains were changed from Brain heart infusion broth (BHI) to Basal defined medium (BDM) for one hour. During osmotic shock, *C. difficile* was shifted from BHI to BHI supplemented with 1.5% NaCl.

### Differential gene expression

**Figure 1:**
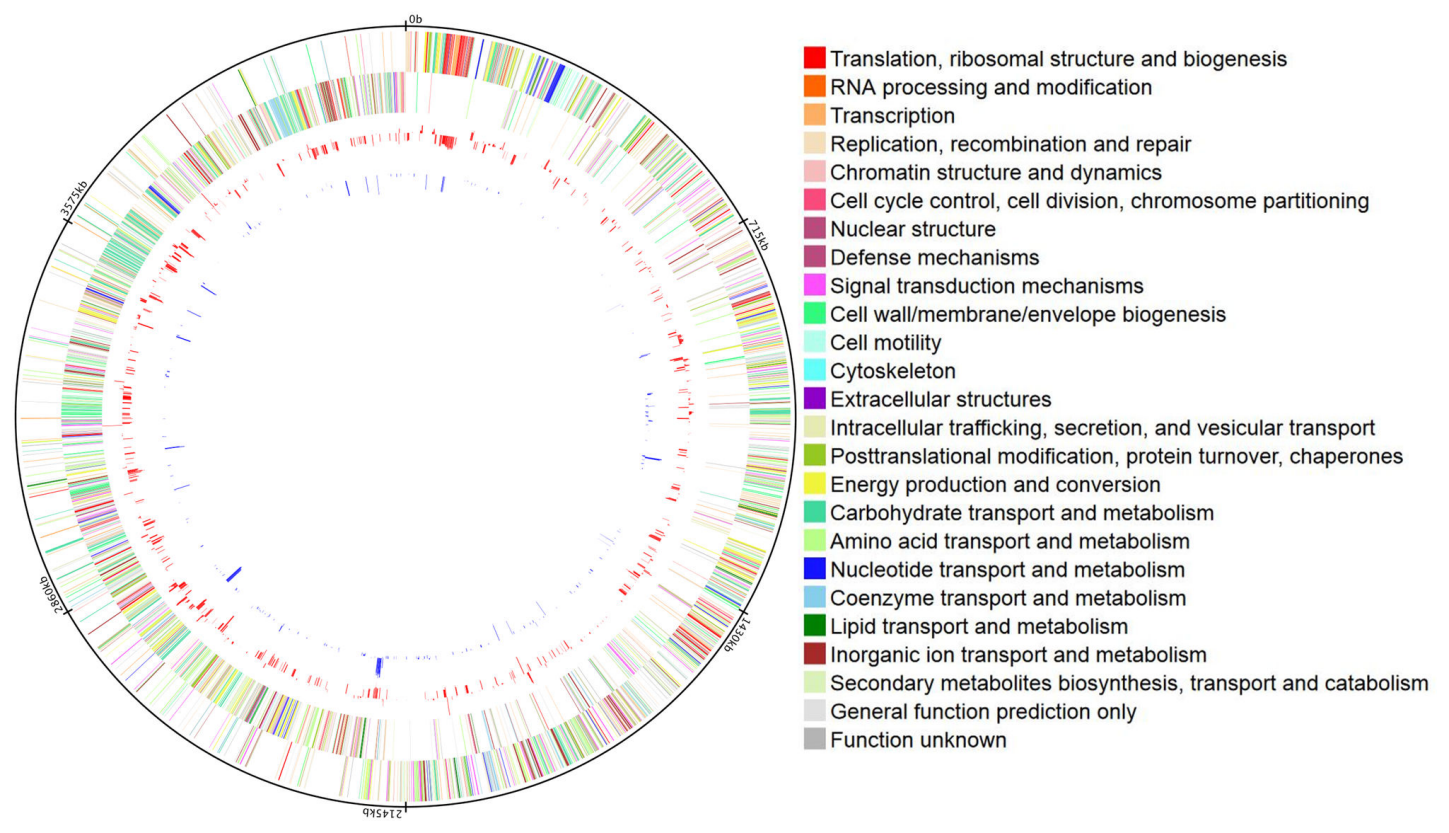
Projection of differentially expressed genes from nutrient shift and osmotic shock on *C. difficile 630* genome. RNA-seq data was converted into FPKM values using cufflinks[44]. When compared to control condition, genes with log ratio ≥ 1.5 and FDR- adjusted *p* value less than or equal to 0.05 were considered as differentially expressed. Circles are numbered from outside to inside. Circle 1 - Molecular clock indicating genome size. Circle 2 - COG gene categories on the forward strand, Circle 3 - COG gene categories on the forward strand. Details of COG color codes in circles 2 & 3 are shown on the right side of the projection. Circle 4 - log_2_ fold change values of differentially expressed genes under nutrient shift. Circle 5 - log_2_ fold change values of differentially expressed genes under osmotic shock.

**Figure 2:**
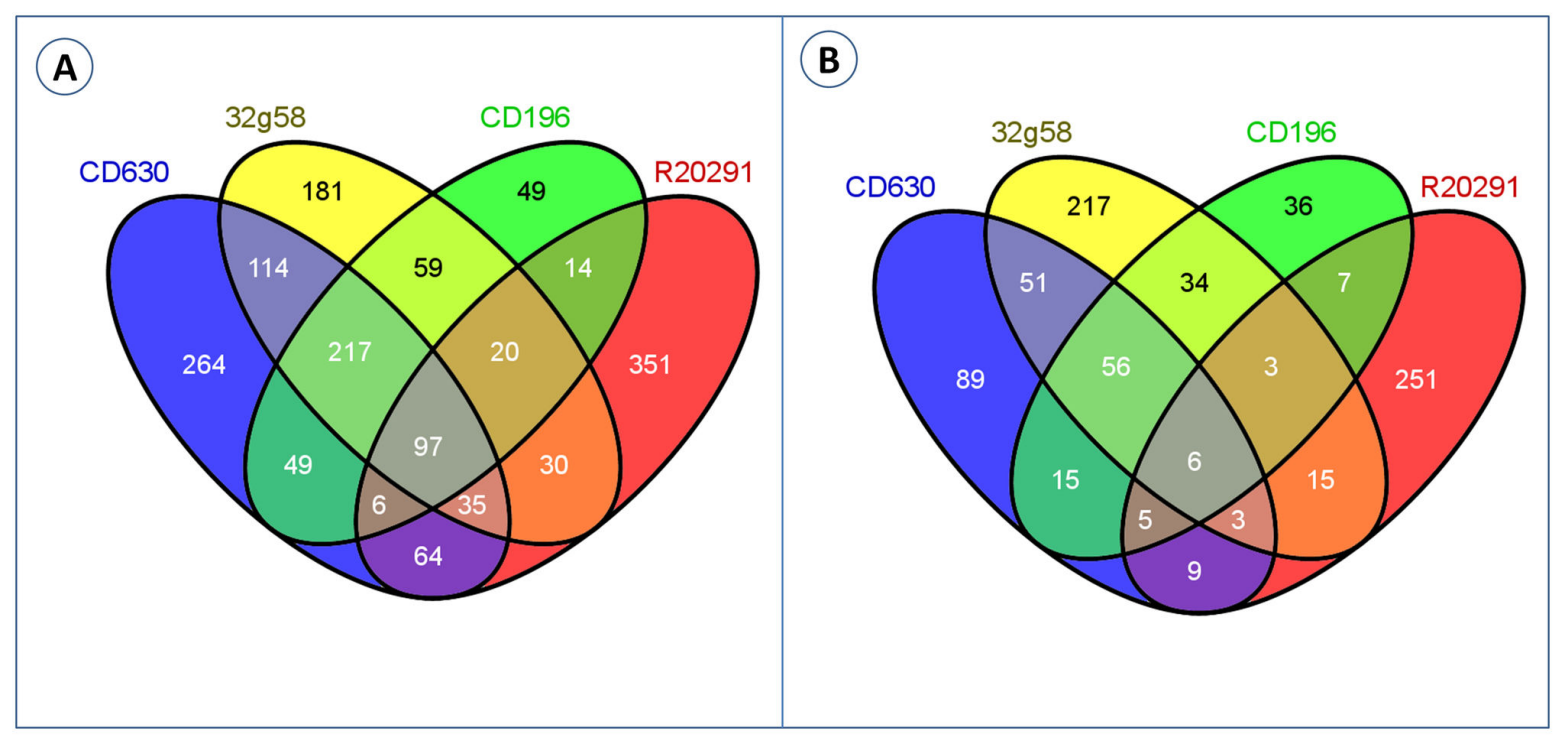
Venn diagram comparing differentially expressed core genes across *C. difficile* strains. Core genes in each strain were determined by applying orthoMCL clustering across publicly available *C. difficile* genomes. When compared to control, genes with log ratio ≥ 1.5 and FDR- adjusted *p* value less than or equal to 0.05 were considered as differentially expressed. Panel A – differentially expressed core genes under nutrient shift (Shift from Brain heart infusion broth to Basal defined medium). Panel B - differentially expressed core genes under osmotic shock(shift from Brain heart infusion broth to Brain heart infusion broth supplemented with 1.5% NaCl).

We used Cuffdiff [36] to calculate differential expression of transcripts. Genes with log_2_ ratio ≥ 1.5 and FDR- adjusted *p* value ≤ 0.05 were considered as differentially expressed. For comparing the differentially expressed genes, we classified genes as core(present in all strains with limited sequence variation), shared (present in some strains) and unique (specific to each strain). Based on orthoMCL clustering of 15 genomes, we had previously defined these gene categories in *C. difficile* [20]. To update these definitions, we added 7 more publicly available *C. difficile* genomes and applied orthoMCL across these 22 genomes. OrthoMCL was run with a BLAST E-value cut-off of 1e-5, 50% identity cut-off, 70% length alignment cut-off and an inflation parameter of 1.5. A total of 7650 clusters were identified in all 22 strains combined. Of these, 2563 were core, 2489 were shared and 2598 were unique. We then combined differentially expressed gene list and orthoMCL gene classification. The number of differentially expressed genes across strains and in each condition is listed in Table 4, and the complete list of differentially expressed genes is given in Files S2 and S3. Nutrient shift caused a greater number of differentially expressed genes as compared to osmotic shock The differentially expressed genes were distributed across COG functional categories and across the *C. difficile* genome (Figure 1). The largest number of differentially expressed genes under nutrient shift belonged to the following COG categories; carbohydrate transport and metabolism (G), amino acid transport and metabolism (E), translation, ribosomal structure and biogenesis (J), energy production and conversion (C), transcription (K), and cell wall/membrane/envelope biogenesis (M). As expected, the highest number of differentially expressed genes during osmotic shock was related to transport functions. These included the following COG categories; amino acid transport and metabolism (E), inorganic ion transport and metabolism (P), carbohydrate transport and metabolism (G), energy production and conversion (C), and cell wall/membrane/envelope biogenesis (M). However, as clearly shown in Table 4, the majority of the differentially expressed CDS were core genes. Surprisingly, only 97 and 6 core genes were differentially expressed across all strains in nutrient shift and osmotic shock respectively (Figure 2). While a subset of differentially expressed core genes overlapped between two or more strains, a very large number of differentially expressed core genes did not show any overlap with other strains. Estimation of expression levels of selected genes using qRT-PCR was in agreement with the trend of fold changes detected using RNA-seq (File S1). The highest number of such differentially expressed non-overlapping core genes was found in strain R20291. Among the core genes differentially expressed across all strains were genes belonging to the phosphotransferase system (PTS). The expression pattern of genes belonging to the PTS system was in agreement with the general properties of PTS. For example, PTS genes associated with utilization of secondary carbon sources such as cellobiose, N-acetylglucosamine, mannose, glucitol, and sorbitol were up-regulated several fold while genes associated with utilization of primary carbon sources such glucose and fructose were highly down-regulated. Ethanolamine genes were also down-regulated. *C. difficile* toxin genes were not among the differentially expressed genes in either condition tested.

**Table 4:** Differential expressed CDS genes and core CDS genes.

**Strain**	**CD630**		**CD196**		**QCD_32g58**		R20291	
**Condition**	**Nutrient shift**	**Osmotic shock**	**Nutrient shift**	**Osmotic shock**	**Nutrient shift**	**Osmotic shock**	**Nutrient shift**	**Osmotic shock**
**Core**	851	234	512	162	765	386	621	301
**Shared**	10	4	27	16	30	41	38	25
**Unique**	27	14	1	0	32	21	1	0
**Total**	888	252	540	178	827	449	660	326

**Figure 3:**
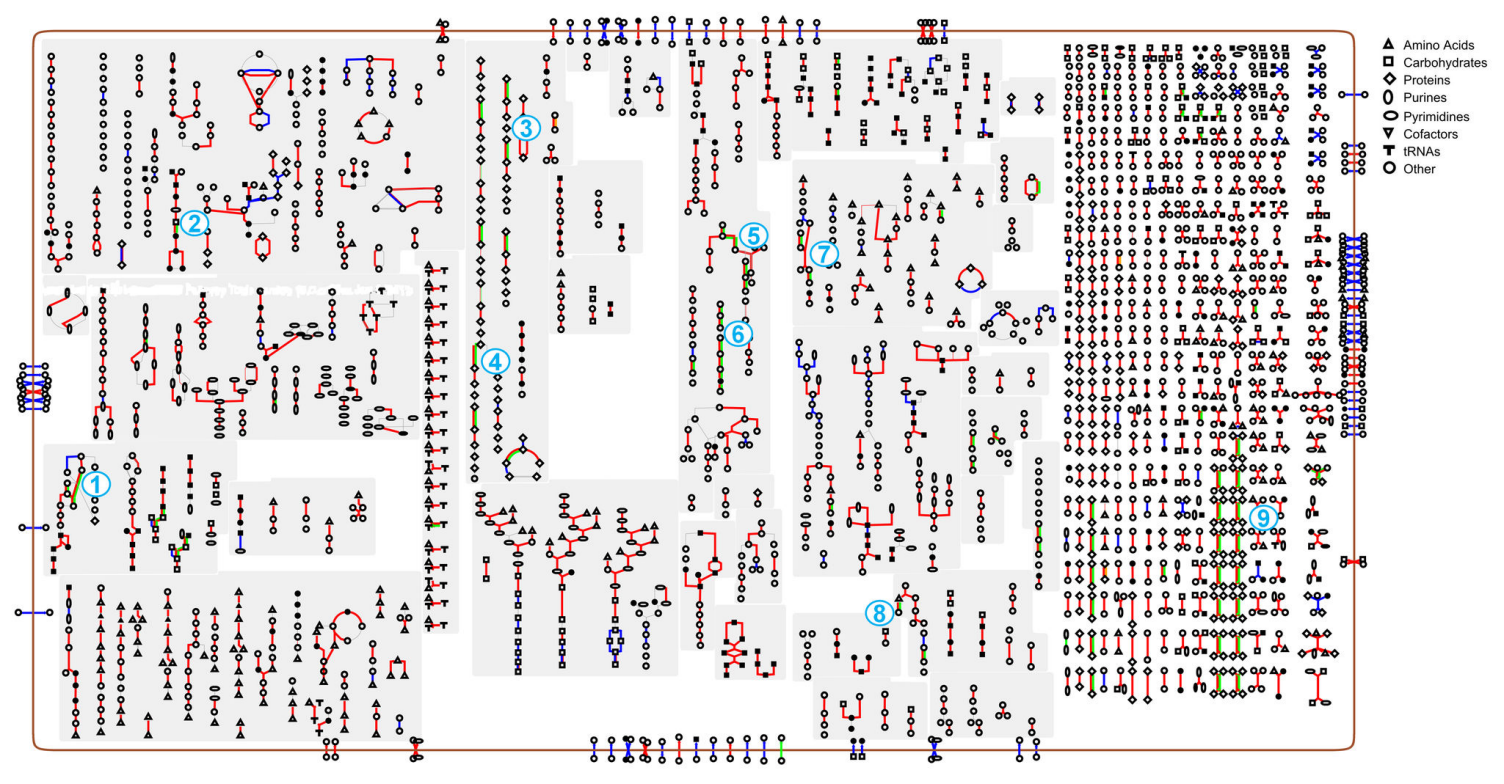
Overview of differentially expressed *C. difficile* pathways. Pathways of *C. difficile* were reconstructed using pathway tools. Amino acids, carbohydrates, proteins, purines, pyrimidines, cofactors, tRNAs and other components in the pathways are coded as per the scheme given on the top right hand side of the figure. Pathways on the left side are biosynthetic pathways, middle are central intermediary metabolism pathways, right side are catabolic pathways and the group on the extreme right are reactions associated with the state of the cell. The following color scheme in the pathways represent; blue - present in at least one strain, red - present in all strains, green - differentially expressed in all strains. Numbers in the pathways denote the following differentially expressed pathways; 1- Gluconeogensis, 2 - Folate transformation, 3- Biotin-carboxyl carrier assembly, 4 - Plamitate biosynthesis, 5 - Pyruvate fermentation, 6 - Fermentation of butanote, 7- Lysine fermentation acetate, 8- Aminobutyrate degradation, 9- Degradation/Utilization/Assimilation.

Since majority of core genes in *C. difficile* are associated with cellular pathways, we analyzed how the tested stress conditions modulate pathways of *C. difficile*. Omics viewer [40] in Pathway tools was used to map differentially expressed genes to cellular pathways. We used enrichment analysis to determine pathways that changed significantly during nutrient shift. Fishers exact test with a p-value of >0.05 was used as the cutoff threshold. This analysis revealed that 20 pathways were significantly enriched in at least one strain (Table 5). Some of the pathways that were differentially expressed in all strains included pathway of gluconeogensis, folate transformation, plamitate biosynthesis and pyruvate fermentation (Figure 3). Using the same *in vitro* conditions used in this study, we have previously reported the proteomic profile of strains compared in this study[43]. There was very good overlap between the number of differentially expressed pathways detected in that study and current results. Some of the differentially expressed genes such as *folD*, *gapA* and *fchA* are multifunctional enzymes associated with more than one pathway. In contrast to nutrition shift, osmotic shock had only minimal impact on pathways. Only six genes (CD0022, CD0079, CD0087, CD0177, CD0627A, CD0628) were differentially expressed across all strains during osmotic shock. Among these, CD0079 and CD0087, which code for ribosomal proteins, were down-regulated. The other four genes, which code for membrane proteins, were up-regulated. 

**Table 5:** List of differentially enriched pathways during nutrient shift.

**Pathway**	**p-value**	**Genes associated**
Carbohydrates Biosynthesis	1.04E-07	**CD0118**, CD0886, CD2318, cooS, eno, fbp, **fchA**, **fhs, folD**, **gapA**, gapB, glgA, glgC, gpmI, pgi, pgk, pmi, pyc, pykF, rkpK, tpiA
Sugars Biosynthesis	3.21E-07	**CD0118**, CD0886, cooS, eno, fbp, **fchA, fhs**, folD, **gapA**, gapB, gpmI, pgi, pgk, pmi, pyc, pykF, rkpK, tpiA
Gluconeogenesis	8.20E-07	**CD0118**, cooS, eno, fbp, **fchA**, **fhs**, folD, **gapA**, gapB, gpmI, pgi, pgk, pyc, pykF, tpiA
Generation of Precursor Metabolites and Energy	1.01E-05	**abfD**, abfH, abfT, adhE, adhE, bcd2, **buk**, cat1, **CD0118**, **CD0715**, **CD2379**, crt2, ctfB, eno, fbp, **gapA**, gapB, gpmI, hydA, pfkA, pgi, pgk, plfB, **ptb**, pykF, rpe, rpiB1, rpiB2, thlA1, tkt, tkt', tpiA
Adenosine nucleotides *de novo* biosynthesis	4.81E-05	adk, atpA, atpB, atpC, atpD, atpF, atpG, atpH, atpI, ntpA, ntpB, ntpC, ntpD, ntpE, ntpK, purA
Fermentation	1.82E-04	**abfD**, abfH, abfT, adhE, adhE, bcd2, **buk**, cat1, **CD0118**, **CD0715**, **CD2379**, crt2, ctfB, eno, **gapA**, gapB, gpmI, pgi, pgk, plfB, **ptb**, pykF, rpe, thlA1
Superpathway of glycolysis and Entner-Doudoroff	2.49E-04	eno, fbp, **gapA**, gapB, gpmI, pfkA, pgi, pgk, pykF, tpiA
Amines and Polyamines Degradation	9.96E-04	**abfD**, abfH, abfT, bcd2, CD1585, **eutB**, eutC, gabT, **gluD**, nanA, nanE, sucD
Purine Nucleotide Biosynthesis	0.001257477	adk, atpA, atpB, atpC, atpD, atpF, atpG, atpH, atpI, CD0489, hpt, iunH, ntpA, ntpB, ntpC, ntpD, ntpE, ntpK, purA, pyrH, pyrR, upp, xpt
Aspartate superpathway	0.002091068	aspD, CD1339, CD2382, CD2828, metE, nadA, nadB, nadC
C1 Compounds Utilization and Assimilation	0.002151781	**CD0118**, cooS, **fchA**, **fhs**, folD, plfB
4-Aminobutyrate Degradation	0.002258078	**abfD**, abfH, abfT, bcd2, gabT, **gluD**, sucD
Pyruvate Fermentation	0.004278705	adhE, adhE, bcd2, **buk**, cat1, **CD0118**, **CD0715**, **CD2379**, crt2, ctfB, plfB, **ptb**, thlA1
Reductive acetyl coenzyme A pathway	0.005518962	cooS, **fchA**, **fhs**, **folD**
Heterolactic fermentation	0.005744886	adhE, adhE, eno, **gapA**, gapB, gpmI, pgi, pgk, pykF, rpe
4-aminobutyrate degradation	0.006648169	**abfD**, abfH, abfT, bcd2, gabT, **gluD**
Folate transformations	0.006927081	CD3456, **fchA**, **fhs**, **folD**, glyA
Methionine Biosynthesis	0.007449931	aspD, CD1339, CD2382, CD2828, dapG, hom2, lysC, malY, metE
Purine Nucleotide *de novo* Biosynthesis	0.009883003	adk, atpA, atpB, atpC, atpD, atpF, atpG, atpH, atpI, CD0489, ntpA, ntpB, ntpC, ntpD, ntpE, ntpK, purA
Degradation/Utilization/Assimilation	0.012817157	**abfD**, abfH, abfT, adhE, adhE, aspD, bcd2, cat1, **CD0118**, CD0723, **CD1339**, CD1585, CD2318, CD2382, CD2819, CD2828, CD3477, cooS, crt2, ctfB, **eutB**, eutC, **fchA**, **fhs**, **folD**, fruK, gabT, garR, glgP, glpK1, glsA, **gluD**, glyA, grdC, grdD, grdE, gutD, mdeA, nanA, nanE, pgmB, phnA, plfB, pmi, prdA, **prdF**, rluB, sucD, thlA1

Genes in bold were also found to be differentially expressed in our previous proteomics study.

**Figure 4:**
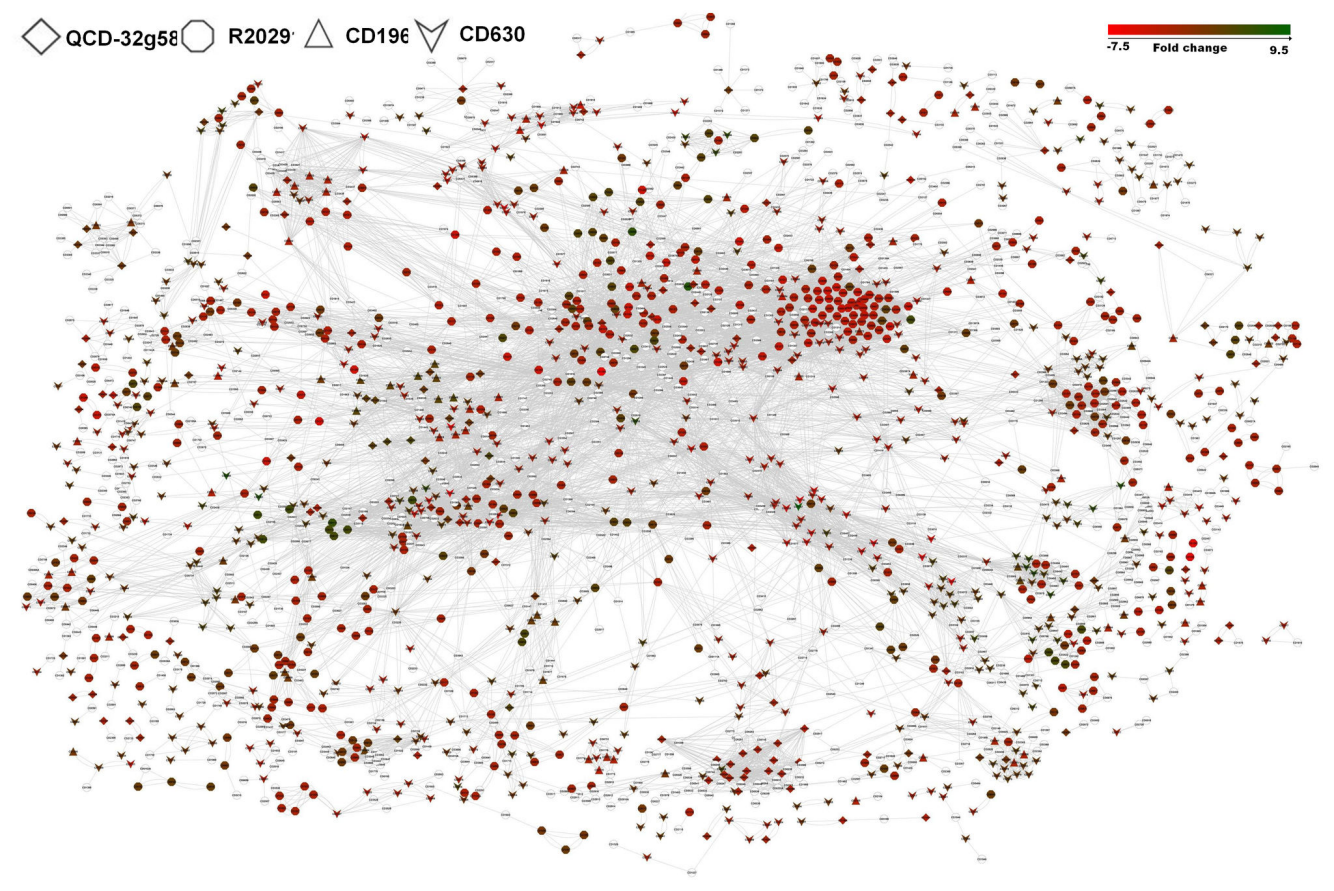
Differentially expressed genes under nutrient shift mapped to *C. difficile* interaction network. The shapes of the nodes denote strains as per the scheme given on top left hand side. The color of the code represents expression fold change. Scale of the fold change is given on top right hand side of the figure.

Pathways in a microbial cell are interconnected to form a system wide interaction network. Subsections of the system wide network control bacterial cell response to physiological changes. To determine the gene interaction network that is activated during nutrient shift, we mapped the differentially expressed genes to *C. difficile* interaction network. The system wide interaction data for *C. difficile* was downloaded from STRING[41]. We then extracted interaction data that were associated with nutrient shift in all strains. This network contained 2149 nodes (genes) and 14186 edges (interactions). A complete list of these nodes and interaction confidence scores is given in File S4. When differential expression data was overlaid on this network, we found that up-regulated and down-regulated genes were scattered all over the network (Figure 4). However, the mean S. P. betweeness of node couples in strain R20291 specific interaction clusters were higher than other strains. 

### Novel gene discovery

**Figure 5:**
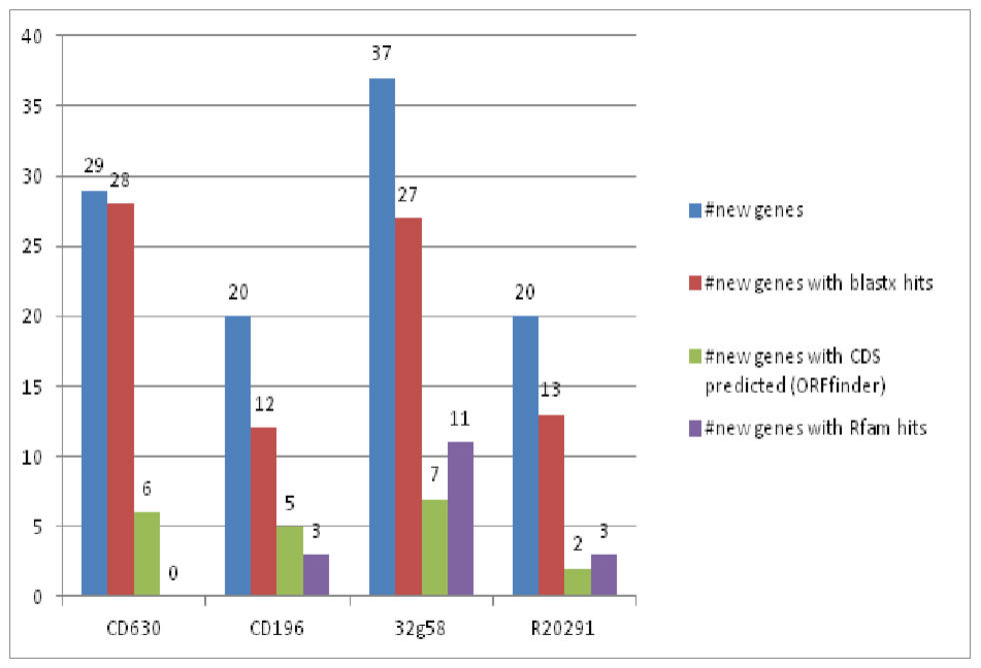
Summary of new genes and potential small RNAs identified in *C. difficile* strains.

Cufflinks assembles transcripts and maps them to the annotated genes of a genome.  If the transcripts do not map to known genes of that genome, then these transcripts could be novel genes. After assembly to known annotated genes, we identified a total of 106 new gene transcripts in the intergenic regions of the four strains compared (File S5). These transcripts could be genes that were missed during genome annotation or could be novel small RNA genes. To determine this, first we searched the Rfam database for sRNA that matched these novel transcripts. Then using BLASTX, we searched non-redundant (nr) database with the new transcripts to check for any protein coding gene hits. We also made ORF predictions using the ORFfinder program to see if any transcripts are potentially protein coding genes. A total 80 transcripts had significant BLASTX hits with evalue cutoff of 0.001 (File S6). For the transcripts that do not have ORFs, they are likely to be sRNAs. The results are summarized in Figure 5. There were a total of 17 genes that matched Rfam small RNAs. Ten of them matched RF01327 (CRISPR-DR14), three matched RF01051 (GEMM_RNA_motif), two matched RF01786 (c-di-GMP-II), one matched RF00230 (T-box), and one to RF00504 (Glycine). 

## Discussion

In the recent years*, C. difficile* has emerged as emerged as a serious human pathogen. Very high genome diversity among *C. difficile* strains is a contributing factor towards this [18,19,25,26]. The core genome content of *C. difficile* is less than 20% of the pangenome[20]. This core genome content is unusually low because core genome of other highly divergent pathogens such as *Streptococcus agalactiae* is at least 80% of the pangenome [46,47]. Despite this massive genome diversity, functional genomics studies in *C. difficile* have mostly been done using strain 630, which is a historic strain isolated from a human patient in the 1980s[21-24]. Recently isolated strains of *C. difficile* are more infectious and virulent than old strains[25,48]. To determine how these old and recent isolates adjust to physiological stress and to provide a better definition of *C. difficile* transcriptome across strains, we performed *in vitro* transcriptome profiling of two old and two recent hypervirulent strains. Nutritional shift from brain heart infusion broth (rich medium) to basal minimal medium was chosen as the first *in vitro* stress condition. This was chosen because *C. difficile* toxins are known to be up-regulated several fold when the growth medium contains difficult to metabolize carbon and nitrogen sources[29,49]. In the second stress condition, cells were shifted to BHI supplemented with 1.5% NaCl to induce osmotic shock. This was selected as the second test condition because *C. difficile* has been shown to have enhanced host cell adherence following osmotic shock [31,32,50]. We used Illumina Hiseq 2000 based RNA-seq to sequence the *C. difficile* transcriptome from these test conditions. When compared to microarray based transcriptome analysis, RNA-seq offers several advantages such as not being limited to detecting transcripts that correspond to existing annotations, low or no background signal, a very large dynamic range and very high reproducibility[51]. Consistent with this, under both *in vitro* conditions tested, we detected transcripts for more than 90% CDS(Table 3). This provides much higher resolution than the results we obtained in our previous microarray based transcriptome analysis of *C. difficile*[22,23]. 

When differentially expressed genes were classified as core, shared and unique based on orthoMCL ortholog clustering, we find that the majority of the differentially expressed CDS were core genes(Table 4). Since each *C. difficile* strain contains a large number of shared and strain specific genes, the low level of differential expression we detected in this study is surprising. This could be because many of the strain specific genes are virulence factors or antibiotic resistance genes that provide niche adaptation[18,25]. These genes may not have been expressed in the conditions tested in this study since nutritional switch and osmotic shock are basic metabolic functions. When the differentially expressed core genes were compared across strains (Figure 2), there was not much overlap between the genes. A total of 97 and 6 core genes were differentially expressed across all strains during nutrient shift and osmotic shock respectively. It is widely known that nutritional availability in the medium determines the amount of toxin produced by various *C. difficile* strains[52-57]. In *C. difficile*, a cluster of genes known as the pathogenicity locus (PaLoc) contains toxin A and B genes and three accessory genes, including *tcdD* and *tcdC*, which are thought to code for the positive and negative regulators of toxin expression, respectively[58]. Little is known about how other genes in individual *C. difficile* strains interact with the genes in PaLoc. The large number of uniquely expressed core genes we found in this study could be a mechanism by which individual *C. difficile* strain adjust to nutrient changes and produce variable levels of toxins. The number of differentially expressed genes during osmotic shock was less than that of nutrient shift. Differentially expressed genes following osmotic shock included genes such as *GroEL, RecA, CspG*, and *CspF*. This is consistent with previous findings that genes such as *GroEL* are up-regulated during osmotic shock and increases *C. difficile* adhesion to host cells[32,50,59]. 

To understand how nutritional shift modulates cellular pathways, differentially expressed genes were mapped to *C. difficile* pathways. Enrichment analysis revealed that 20 pathways were significantly changed during nutrient shift (Table 5). Using the same strains used in this study, we had previously reported the proteome profile of *C. difficile* during nutrient shift and osmotic shock[43]. Pathways found to be differentially expressed at the protoemics level correlated well with RNA-seq results(Table 5). However, the number of differentially expressed genes detected using RNA-seq was larger when compared to the number of pathways detected at the proteome level. This could be due to the differences in the resolution of the technologies and the depth of sequencing. The number of transcripts that can be detected in one single lane RNA-seq run is far more than the number of peptides that can be detected using TMT based nanoLC–MS/MS proteome sequencing. In this study, each cDNA library was sequenced using one full flow cell without multiplexing. In our previous proteomics study, four samples were multiplexed in one sequencing reaction. These factors could account for the differences in the number of differentially expressed genes detected in both studies. 

Despite the differences in the number of genes detected at the transcriptome and proteome levels, a number core functions that control metabolism were common in both data sets. Phosphotransferase system (PTS) is composed of a cluster of genes that regulate the switch between utilization of primary and secondary carbon sources [60-62]. Several genes belonging to PTS were upregulated in both the in RNA-seq as well as proteome sequence data sets. In contrast, ethanolamine utilization genes were found to be down-regulated in both datasets. Members of the genus *Clostridium* can use ethanolamine as a source of carbon or nitrogen[63]. Host diet and gut epithelial cells are an important source of ethanolamine for bacteria[63,64]. Although the defined medium we used in this study does not contain any ethanolamine, BHI contains large amount of animal cell derived nutrients. This could contribute significant amounts of ethanolamine in BHI and the observed down-regulation of ethanolamine proteins could be a result of this concentration difference.

When differentially expressed genes under nutrient shift were overlaid on the *C. difficile* gene interaction network, the differentially expressed genes from all four strains were scattered across the network(Figure 4). However, when compared to other strains, the average S. P. betweeness of the node couples differentially expressed in strain R20291 was more than other strains. The S. P. betweeness is a node centrality index. The S. P. betweenness of a node in a biological network is an indicator of the relevance of a protein’s functional capability to hold communicating proteins together[65]. The S. P. betweenness of a protein effectively indicates the capability of a protein to recruit? distant proteins[65]. Strain R20291 is a hypervirulent strain associated with a severe outbreak and several mortalities in Aylesbury, UK[25]. Although it produces very low numbers of spores[66], it is highly infectious[25]. A more efficient gene interaction network indicated by increased S. P. betweeness in node pairs of R20291 might enable this strain to respond to environmental changes more efficiently. This could translate to better survival within a host. 

One of the advantages of the RNA-seq technology is the ability to detect transcription of genes that are not part of the primary genome annotation[51]. Consistent with this, we detected a number of transcriptionally active regions in all four *C. difficile* strains that were not part of the genome annotation of the respective strain (File S5). BLASTX searches showed that the majority of these assembled transcripts were annotated as protein coding genes in other *C. difficile* genomes or other bacterial species (File S6). This shows that RNA-seq could detect genes that were missed by microbial annotation engines and can be used to improve the genome annotation. When searched against Rfam database, some of the transcriptionally active regions did match small RNAs. A recent study has found that small noncoding RNAs (sRNAs) in *C. difficile* are involved in the regulation of motility and biofilm formation[67]. Therefore, the small RNAs detected in this study could be involved in the regulation of nutritional switch and osmolarity sensing in *C. difficile*. Further work is required to ascertain this possibility. 

## Supporting Information

File S1 **Details of primers used for qPCR and comparison of qPCR and RNA-seq data for select genes.**
(XLSX)

File S2 **Details of differentially expressed genes during nutrient shift.** Data for strains QCD-32g58, R20291, CD196 and CD630 are given in separate worksheets named after these strains.
(XLSX)

File S3 **Details of differentially expressed genes during osmotic shock.** Data for strains QCD-32g58, R20291, CD196 and CD630 are given in separate worksheets named after these strains.
(XLSX)

File S4 **Protein interaction data for *C. difficile* genes involved in nutrient shift.** : Nodes are represented by NCBI locus tags for the CDS. The combined interaction score represents cumulative interaction score for each edge.
(XLSX)

File S5 **Details of new gene transcripts identified among the compared strains.**
(XLSX)

File S6 **Details of BLASTX searches for the new gene transcripts.**
(XLSX)
